# Cardiovascular health among the Czech population at the beginning of the 21st century: a 12-year follow-up study

**DOI:** 10.1136/jech-2017-209967

**Published:** 2018-02-08

**Authors:** Michala Lustigova, Dagmar Dzurova, Hynek Pikhart, Ruzena Kubinova, Martin Bobak

**Affiliations:** 1 Environmental and Population Health Monitoring Centre, National Institute of Public Health, Prague, Czech Republic; 2 Department of Social Geography and Regional Development, Faculty of Science, Charles University, Prague, Czech Republic; 3 Department of Epidemiology and Public Health, University College London, London, UK

**Keywords:** epidemiology of cardiovascular disease, lifestyle, mortality, cohort studies, demography

## Abstract

**Background:**

In the late 1980s, Czechia was among the countries which had the highest cardiovascular mortality in the world. In spite of enormous improvements since that time, there are still large opportunities in further improving cardiovascular health.

**Methods:**

Based on the Czech Health, Alcohol and Psychosocial Factors in Eastern Europe sample (n=8449 at baseline, 12 years of follow-up, 494 cardiovascular disease (CVD) deaths up to 2015—events), the impact of selected covariates such as education, smoking habits, high blood pressure, blood cholesterol level, diabetes, obesity, physical activity and binge drinking and their multifactorial effects on cardiovascular mortality was evaluated by Cox regression. In addition, population attributable fractions (PAFs) were used to quantify the impact of these factors on CVD mortality in the population.

**Results:**

Education was found as the strongest determinant of CVD mortality (primary vs university, HR 2.77, P<0.001; PAF=50.5%). CVD risk was two times higher for persons with diabetes compared with those without (HR 2.02, P<0.001, PAF=23.2%). Furthermore, significant factors found were smoking (smoker vs non-smoker, HR 1.91, P<0.001; PAF=26.5%), high blood pressure (HR 1.73, P<0.001; PAF=35.3%) and physical inactivity (none vs sufficient, HR 1.60, P<0.001; PAF=22.9%). Conversely, the effect of obesity was low (HR 1.29, P value =0.020), and binge drinking and high blood cholesterol level were not significant at all.

**Conclusions:**

Education had the largest impact on cardiovascular mortality among the Czech population. More than 50% of CVD death would be prevented if the whole population had the same risk values as the highest educated population. Reducing disparities in health related to education should benefit from attention to cardiovascular health literacy.

## Background

**Figure 1:**
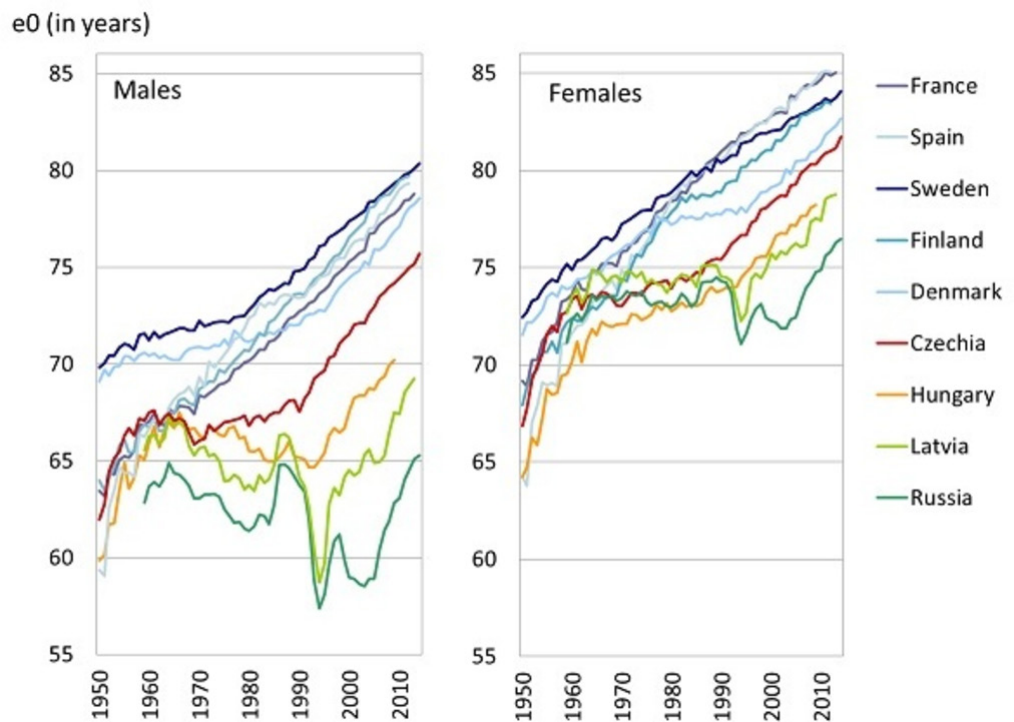
Trends in life expectancy at birth (e0) in selected European countries, 1950–2014. Data source: Human Mortality Database. University of California, Berkeley, USA, and Max Planck Institute for Demographic Research, Germany. Available at www.mortality.org or www.humanmortality.de (data downloaded on 5 January 2016).

In the late 1980s, Czechia, similarly to other postcommunist countries, had extremely higher cardiovascular mortality compared with Western European countries. It was a result of the different population health development from 1960s. While the Western Europe experienced progress in life expectancy thanks to new advances in the treatment of cardiovascular diseases (CVDs) (cardiovascular revolution), the east countries, then governed by Communist regimes, were struck by a health crises, which in some cases resulted even in life expectancy decline, especially among males (see figure 1).

At the end of 1980s, the rapid transition from socialists economic to market economic caused further divergence in mortality. While in some countries like in Czechia, rapid improvement in health condition were recorded and belatedly cardiovascular revolution took place; others were struck by deep mortality crisis and sharp increase in mortality (eg, Baltic States). That initial deterioration in level of mortality was replaced in the late 1990s by the improvement; however, this turmoil brings extra deepening of differences in mortality profiles between European populations.

**Figure 2:**
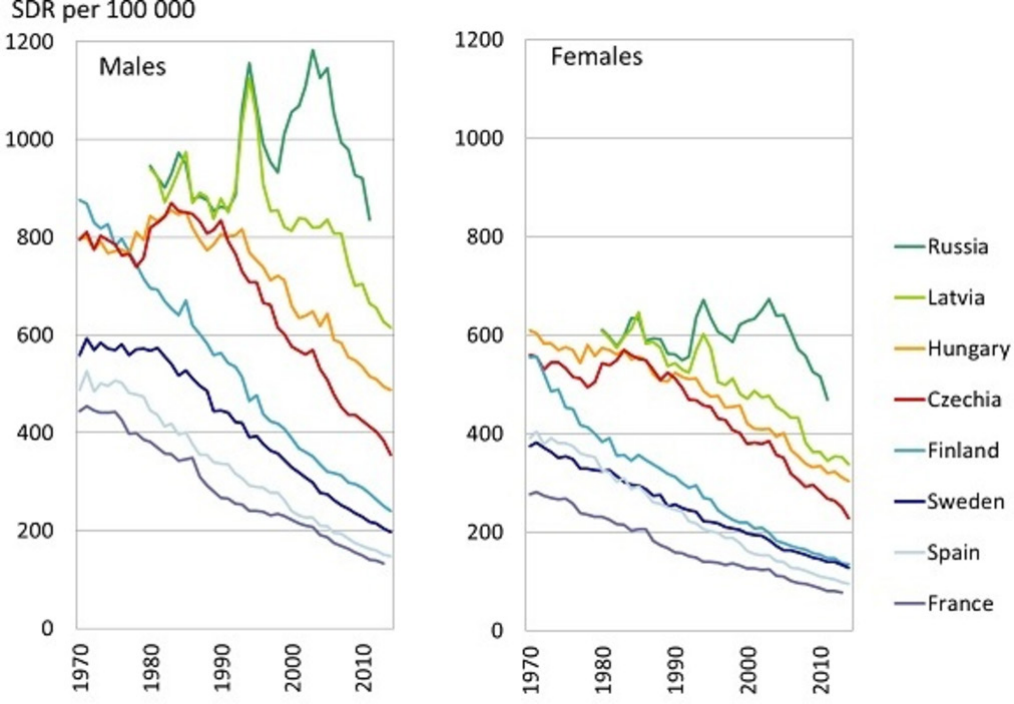
Trends in mortality due to circulatory system diseases (standardised death rate (SDR) per 100 000 inhabitants) in selected European countries, period 1970–2014. Data source: WHO European Health for All database, August 2016.

Since 1990s, among the Czech population, many changes and enormous improvements in cardiovascular health have occurred and resulted in more than 50% decline of CVD mortality rates; both due to changes in lifestyle, subsequent decrease in prevalence of risk factors and due to changes in healthcare.^1–3^ This trend diverted Czechia from most of the Eastern European countries closer to Western European countries^4^ (see figure 1); however, the east–west mortality gap still persists in the case of CVDs (see figure 2). It is mainly the mortality due to ischaemic heart disease (IHD) (International Classification of Diseases 10th revision (ICD-10), dg. I20–I25) which causes the differences. The level of IHD mortality in Czechia (standardised death rate (SDR) in 2014 was 199 per 100 000 males, 115 per 100 000 females) is more than two times higher than in the European Union (EU) (SDR in 2014 was 96 per 100 000 males, 47 per 100 000 females). On contrary, the level of mortality due to stroke (ICD-10, dg. I60–I69) in Czechia (SDR in 2014 was 61 per 100 000 males, 48 per 100 000 females) is only about 25% higher than in EU (SDR in 2014 was 49 per 100 000 males, 39 per 100 000 females).

While the countries of Western Europe have passed the stage of cardiovascular revolution predominantly due to changes in lifestyle,^1 5 6^ the Czech population still remains far from having a ‘healthy lifestyle’, mainly due to a diet rich in saturated fat and salt and poor in fruit and vegetable intake,^2 7^ lack of physical activity leading to overweight and the prevalence of diabetes.^8^ Changes in the lifestyle of the Czech population are very slow compared with the extraordinary improvements in healthcare made during the 1990s.^1^

The goal of this paper is to evaluate cardiovascular risk among the Czech population, quantify the impact of main risk factors on cardiovascular mortality and to identify potential modifiable factors for improving the cardiovascular health.

## Data and methods

### Study subjects

We used Czech data from the Health, Alcohol and Psychosocial Factors in Eastern Europe (HAPIEE) longitudinal study. The study was set up to investigate determinants of mortality in Central and Eastern European populations. In 2002–2005 (baseline survey), 8449 persons aged 45–69 years were randomly selected from the population register (of involved six Czech cities into study), stratified by sex and 5-year age bands. At the baseline, the Czech participants completed a structured questionnaire at home and then were invited to a medical examination in a clinic (complete medical examination with blood collection and cognitive and physical tests). Overall response rate was 54% in the questionnaire survey; from those who answered the questionnaire, 82% participated in the medical check-up. The cohort has been regularly monitored for changes in health status (repeated postal questionnaire sent to still participating respondents every 2 years) and mortality. Deaths in the baseline cohort were identified through the mortality register. There were 1284 deaths in the cohort within the monitored period (2002–2015). All participants gave their written informed consent. A detailed description of the study is provided elsewhere.^9^

### Covariates

We used the information from the baseline survey and from the Czech mortality register on respondents’ deaths. The basic model included sociodemographic characteristics such as age, sex, education and marital status. Age was considered to be a completed age at the baseline. Marital status was dichotomised into married/cohabiting versus others. Educational attainment contained four groups: university, secondary, vocational and primary (or incomplete) education.

Among CVD risk factors, the impacts of smoking habits, high blood pressure, blood cholesterol level, diabetes, obesity, physical activity and binge drinking were evaluated. Smoking habits included three categories: non-smoker, ex-smoker and smoker. Obesity (body mass index (BMI) ≥30 kg/m^2^), high blood pressure (BP ≥140/90 mm Hg), self-reported diabetes were dichotomised covariates. Binge drinking was defined at threshold 100+g of ethanol at least monthly. The criterion was the same for males and females and responds to widely used binge drinking definition of five and more drinks per one occasion.^10^ Physical activity was categorised as none, insufficient (1–3 hours a week) and sufficient (four and more hours a week). Categories were defined on the basis of recommendation of ACSM/American Heart Association from 1995,^11^ and the threshold for sufficient physical activity was 3.5 hours.

And finally blood cholesterol level (total cholesterol (TC)) was categorised as low risk (TC below 5.0 mmol/L), increased risk (TC 5.0–6.2 mmol/L) and high risk (TC above 6.2 mmol/L). Used cholesterol stratification was based on the combination of two recommendations—recommendation of American National Heart, Lung and Blood Institute (NHLBI) from 2002^12^ and the European guideline on CVD prevention.^13^ Low risk (TC <5.0 mmol/L) category was based on European guidelines and the high risk category was based on NHLBI recommendation (TC above 240 mg/dL).

Blood pressure, blood cholesterol level and obesity were based on medical examination. Remaining variables were based on the subjective responses in questionnaires. As all the variables were categorised, the missing values were treated as a single category (number of not available or missing values is visible in table 1). The HRs for those categories are not displayed in the tables 2 and 3. Furthermore, all models were controlled for the impact of respondent’s self-reported history of CVD at the baseline (ischaemic heart disease or stroke) as a dichotomised covariate.

**Table 1:** Distribution of sociodemographic characteristics and cardiovascular disease risk factors among the HAPIEE cohort at the baseline, 45–69 aged, Czechia, 2002–2005

		HAPIEE cohort		Died in HAPIEE cohort due to CVD*		The rest of HAPIEE cohort		P value for the difference between died due to CVD and the rest of the HAPIEE cohort†
		n (8449)	%	n (494)	%	n (7955)	%	
Sociodemographic factors								
Average age (in years)		58.3		63.4		58.0		<0.001
Self-reported history of CVD (ischaemic heart disease or stroke)	No	7331	86.8	309	62.6	7022	88.3	<0.001
	Yes	1118	13.2	185	37.4	933	11.7	
Sex	Male	3955	46.8	314	63.6	3641	45.8	<0.001
	Female	4494	53.2	180	36.4	4314	54.2	
Education	Primary	1060	12.5	105	21.3	955	12.0	<0.001
	Vocational	3135	37.1	205	41.5	2930	36.8	
	Secondary	3061	36.2	143	28.9	2918	36.7	
	University	1150	13.6	34	6.9	1116	14.0	
	n. a.‡	43	0.5	7	1.4	36	0.5	
Marital status	Married/cohabiting	6366	75.3	334	67.6	1892	23.8	<0.001
	Single/divorced/widowed	2047	24.2	155	31.4	6032	75.8	
	n. a.‡	36	0.4	5	1.0	31	0.4	
Metabolic and lifestyle factors								
Smoking habits	Smoker	2194	26.0	143	28.9	2051	25.8	<0.001
	Ex-smoker	2472	29.3	179	36.2	2293	28.8	
	Non-smoker	3673	43.5	163	33.0	3510	44.1	
	n. a.‡	110	1.3	9	1.8	101	1.3	
Obesity (BMI ≥30 kg/m^2^)	Yes	2188	25.9	171	34.6	2017	25.4	<0.001
	No	4941	58.5	198	40.1	4743	59.6	
	n. a.‡	1320	15.6	125	25.3	1195	15.0	
High blood pressure (BP ≥140/90 mm Hg)	Yes	3874	45.9	274	55.5	3600	45.3	<0.001
	No	3241	38.4	91	18.4	3150	39.6	
	n. a.‡	1334	15.8	129	26.1	1205	15.1	
Blood cholesterol level (TC in mmol/L)	Low risk (TC <5.0)	1554	18.4	94	19.0	1460	18.4	<0.001
	Increased risk (TC 5.0–6.2)	2946	34.9	144	29.1	2802	35.2	
	High risk (TC >6.2)	1996	23.6	99	20.0	1897	23.8	
	n. a.‡	1953	23.1	157	31.8	1796	22.6	
Diabetes	Yes	997	11.8	146	29.6	851	10.7	<0.001
	No	7426	87.9	344	69.6	7082	89.0	
	n. a.‡	26	0.3	4	0.8	22	0.3	
Physical activity (in hours per week)	None (0)	2514	29.8	215	43.5	2299	28.9	<0.001
	Insufficient (1–3)	2168	25.7	93	18.8	2075	26.1	
	Sufficient (4+)	3491	41.3	152	30.8	3339	42.0	
	n. a.‡	276	3.3	34	6.9	242	3.0	
Binge drinking (100 g of pure alcohol during one occasion in a month)	Yes	806	9.5	41	8.3	765	9.0	0.143
	No	7352	87.0	429	86.7	6932	87.0	
	n. a.‡	291	3.4	24	4.9	267	3.4	

*Characteristics of respondents who died during the period 2002–2015 due to CVD (ICD-10, dg. I00–I99).

†P value calculated with analysis of variance regression for continuous variable, and comparison of proportions between groups of categorical variable was based on χ^2^ test.

‡Category n. a. covers also the missing values among the respondents.

BMI, body mass index; CVD, cardiovascular disease; HAPPIE, Health, Alcohol and Psychosocial factors in Eastern Europe; ICD, International Classification of Diseases; n.a., not available; TC, total cholesterol.

**Table 2:** Association between risk factors and cardiovascular mortality

Risk factor	HR	95% CI	P value
Diabetes (yes vs no)	2.24	1.84 to 2.73	<0.001
Smoker versus non-smoker	2.07	1.64 to 2.62	<0.001
Physical activity (none vs sufficient)	2.00	1.62 to 2.45	<0.001
High blood pressure (yes vs no)	1.87	1.47 to 2.38	<0.001
Obesity (yes vs no)	1.62	1.32 to 1.99	<0.001
Binge drinking (yes vs no)	1.35	0.90 to 2.03	0.150
Blood cholesterol level (high risk vs low risk)	0.91	0.68 to 1.21	0.504

Controlled for age, sex and self-reported history of cardiovascular disease.

**Table 3:** Association between selected covariates and cardiovascular mortality, basic and final model

Covariates		Basic model			Final model		
		HR	95% CI	P value	HR	95% CI	P value
Sociodemographic factors							
Age (completed)		1.12	1.01 to 1.14	<0.001	1.12	1.10 to 1.14	<0.001
Sex	Female	1		<0.001	1		<0.001
	Male	2.53	2.07 to 3.09		2.07	1.68 to 2.57	
Education	University	1			1		
	Primary	3.70	2.48 to 5.51	<0.001	2.77	1.85 to 4.14	<0.001
	Vocational	2.27	1.58 to 3.27	<0.001	1.93	1.34 to 2.79	<0.001
	Secondary	1.88	1.29 to 2.73	0.001	1.84	1.26 to 2.68	0.002
Marital status	Married/cohabiting	1		<0.001	1		<0.001
	Single/divorced/widowed	1.71	1.32 to 2.09		1.65	1.35 to 2.02	
Metabolic and lifestyle factors							
Smoking habits	Non-smoker				1		
	Smoker				1.91	1.50 to 2.42	<0.001
	Ex-smoker				1.26	1.00 to 1.57	0.047
Obesity	No				1		0.018
	Yes				1.29	1.05 to 1.59	
High blood pressure	No				1		<0.001
	Yes				1.73	1.36 to 2.20	
Diabetes	No				1		<0.001
	Yes				2.02	1.65 to 2.46	
Physical activity	Sufficient				1		
	None				1.60	1.30 to 1.98	<0.001
	Insufficient				1.09	0.84 to 1.41	0.524
Log likelihood		−4110			−4027		
Likelihood ratio test, P value					<0.001		

Models are controlled for self-reported history of cardiovascular disease.

### Statistical analysis

The impact of the above-mentioned covariates on cardiovascular mortality was evaluated using the survival analysis method, specifically Cox regression (a regression model of proportional risks). Testing of the assumption of risk proportionality was performed by a graphic method based on the transformation of estimates of the survival function using the log(−log S(t)) function. Curves were controlled both for age and sex.

The monitored event was cardiovascular death (n=494). A cardiovascular fatal event was defined as a death where the underlying cause was due to diagnoses of I00–I99 in the ICD-10 revision. The number of events by CVD type was followed: 22 cases of hypertension (I10–I15), 225 cases of ischaemic heart disease (I20–I25), 41 cases of sudden death (I50), 84 cases of stroke (I60–I69), 36 cases of atherosclerosis (I70) and 86 of other CVD cases. All other deaths (n=790) representing a so-called competing risk were censored at the day of death. Survival time was monitored in terms of months and was defined as follows: the process time began at the moment of entry into the study and the end of process time was the moment of death or the time when the respondents were censored (in 31 December 2015). The proportional risk (HR) expresses how many times the risk of a fatal cardiovascular event for a given category is higher or lower compared with the reference category (with the risk value equal to 1).

The population at risk was made up of a group of 8449 individuals. The process time of individuals in the group ranged from 1 to 167 months with the median time of 146 months and the total of 1 172 781 person-months. Explanatory covariates were considered to be time constant (as observed at the baseline).

### Models

The analysis was done in a few steps. First, the impact of CVD risk factors was estimated separately. Each factor was added in the model controlled just for age, sex and history of CVD as a single variable. Then the basic model including the sociodemographic characteristics and history of CVD was constructed. Finally, the covariates were added one by one into the basic model. Model selection was made using iteration—the model was compared with the closest preceding model (the one that included with each single iteration one factor less) using the Likelihood ratio test. In the final model, only the covariates with significant effect on cardiovascular mortality as well as variables with significant effect on the model were left.

Population attributable fractions (PAFs) were finally estimated for covariates left in the final model. PAFs can qualify the contribution of a risk factor to a death. In our case, the PAFs present proportional reduction of cardiovascular deaths if exposure to a risk were reduced to the lowest observed exposure scenario (eg, the whole population has the same risk as highly educated individuals). PAFs (%) could be understood as the portion of deaths that would be prevented if the whole population had the same risk of death as the reference group. Furthermore, the PAFs could help to estimate which factor has a stronger impact on cardiovascular health in the population. PAFs for individual risk factors can overlap 100% as a result of interaction of impact of individual risk factors on CVD deaths as these deaths are caused by multiple risk factors.

The PAFs were computed only for the modifiable risk factors from the final model.

Statistical software Stata V.12 was used to process and evaluate the data.

## Results

Table 1 presents the distribution of basic characteristics and CVD risk factors among the Czech HAPIEE cohort at the baseline (n=8449) and further separately among those who already died from CVD during the period 2002–2015 (n=494) and the rest of the cohort (n=7955). The differences between died due to CVD and the rest of the HAPIEE cohort are displayed too. Statistically significant differences between these two subcohorts were found for all covariates except the binge drinking prevalence.

The distribution of risk factors among the middle-age population of HAPIEE study corresponds to findings from other sources, that is, findings of European Society of Cardiology,^8^ where the Czechia belongs to the countries with highest prevalence of raised blood pressure, raised blood glucose, diabetes and obesity. On the other hand, the prevalence of raised blood cholesterol has reached the European average^8^; even it was enormously high in Europe at the beginning of 1990s.^14^ Smoking epidemic was never so spread as in Eastern European countries; so the prevalence of smoking among the Czech population is more similar to population in central Europe such as Germany or Austria.^8 15^

### Effect of risk factors separately

Associations between risk factors separately and cardiovascular mortality are presented in the table 2. The proportional risk (HR) is displayed only for the highest risk category compared with the reference category (the lowest risk category). The highest increased cardiovascular risk was found for individuals with diabetes compared with those without (HR 2.24), further for smoking (HR 2.07), physical inactivity (HR 2.00) and high blood pressure (HR 1.87). Obesity was still a statistically significant factor, 1.62 higher risk for obese versus non-obese. On the contrary, the effect of blood cholesterol level and the effect of binge drinking in HAPIEE cohort were not significant.

### Basic model

All selected sociodemographic characteristics included in the basic model (see table 3) were found as significant covariates influencing the cardiovascular mortality. The highest differences were found among educational attainment. Those with primary education were nearly in 3.7 times higher risk compared with those with university education. Risk was also higher among secondary and vocational education (about two times higher). Unmarried persons had increased risk by 71% compared with married and cohabiting ones. The cardiovascular risk increased significantly with age, by 12% per year of age. Risk was also significantly higher (2.53 times) for males than for females.

### Final model

Even in the final model (see table 3), education was found as one of the strong determinants of CVD mortality. The primarily educated had a 2.77 times higher risk compared with the university education. As for metabolic and lifestyle factors, the mortality risk was two times higher for persons with diabetes compared with those without and nearly two times higher for smokers (smoker vs non-smoker). Similarly, an impact was found for high blood pressure (yes vs no) and physical inactivity (none vs sufficient), higher risk by 73% and 60%, respectively. Results moreover showed that quitting smoking reduces the cardiovascular risk; the risk is much lower than among smokers, however, still persists compared with non-smokers (HR 1.26, P=0.047). Further, the model showed that insufficient physical activity did not increase the risk compared with sufficient physical activity (HR 1.09, P=0.524). Conversely, the effect of obesity was low (HR 1.29, P=0.020), and binge drinking and high blood cholesterol level were not significant at all.

### Population attributable fraction

Education was the strongest determinant of CVD health among the Czech population; more than 50% of deaths would be reduced if the whole population had the same risk as the population with university education (see table 4). The next strong determinant was high blood pressure. The elimination of that factor in the population would lead to a 35% reduction of CVD deaths. The absence of use of tobacco would reduce 27% of deaths. A more than 20% reduction was estimated for physical activity and diabetes; sufficient physical activity would reduce 23% of CVD deaths as well as hypothetical elimination of diabetes. On the other hand, the impact of obesity on the health of the population was low (in case of obesity, it can be caused by a low difference between risks).

**Table 4:** Population attributable fraction (PAF) of selected covariates in the final model and cardiovascular disease mortality (PAF, %)

Risk factor	PAF (%)
Education (university vs others)	50.5
High blood pressure (yes vs no)	35.3
Smoking (smoker and ex-smoker vs non-smoker)	26.5
Diabetes (yes vs no)	23.2
Physical activity (none and insufficient vs sufficient)	22.9
Marital status (married/cohabiting vs others)	17.0
Obesity (yes vs no)	11.9

## Discussion

In this large prospective cohort study, estimated impact (HRs) of main risk factors on cardiovascular health is largely consistent with the general finding about CVD risk factors; however, their manifestation (PAFs) among the population is quite unique among the Czech population.

Several limitations of the study need to be considered when interpreting the results. First, there were missing values among part of the population, which did not participate in medical check-up. This part of population was left in the analysis and treated as a separate category in the analysis. The second major issue is that the information about the non-fatal events was available only at the baseline of the survey, which could lead to diluting of factor effect on cardiovascular health. Third, combining all of the risk factors in one single model had reduced the effect of particular risk factors. Metabolically linked risk factors are highly correlated and tend to accompany. The analysis, however, did not evaluate the clustering of risk factors in individual. Finally, some of the results might be influenced by self-reported information, especially on lifestyle-related variables such as alcohol intake. On contrary, the results are based on large sample data, enormous set of variables based both on questionnaire and medical examination, 12 years of follow-up and similar studies are very rare in Czechia.

At present, socioeconomic factors are considered to be the most important determinants of health.^16^ Achieved education, which was used as a measure of socioeconomic position, showed that the education is the most significant factor influencing cardiovascular health among the Czech population. According to Organisation for Economic Co-operation and Development,^17^ in Central European countries and Baltic States the highest health gap by education is observed. To be specific, the difference in life expectancy at age 30 between university educated and below upper secondary educated is more than 10 years among the Czech males.^17^ As circulatory problems were revealed as the main factor explaining the mortality gap between educational groups at older age,^18^ such an impact of education on Czech cardiovascular health is not surprising then.

High blood pressure was confirmed as a significant risk factor contributing to cardiovascular mortality increasing the risk by 73% in the final model. INTERHEART study estimated that 22% of myocardial infarction (MI) in Europe is related to hypertension.^19^ According to our findings, the impact of hypertension on cardiovascular health is higher among the Czech population (PAF=35.3%), which verifies one of the highest prevalence of raised blood pressure in Europe.

A negative effect of smoking was confirmed by our results as well. Several studies confirmed that mortality of smokers due to circulation system diseases is at minimum 50% higher compared with non-smokers.^20 21^ The risk was found in our case to be 91% higher for smokers compared with lifetime non-smokers. Moreover, the risk of CVD event was much lower for ex-smokers, which confirmed very quick effect of quitting smoking on cardiovascular risk.^22^ The effect of smoking on cardiovascular health is lower, about 26% (PAF), compared with the findings from INTERHEART study, where the smoking was quantified as responsible for 36.4% of MI death.

The correlation between diabetes and CVD has been known for a long time. For example, the Framingham study estimated the risk of clinical development of atherosclerotic disease to be two to three times higher in patients with diabetes compared with persons without diabetes.^23^ The negative effect of diabetes on CVD mortality was confirmed also by data from the HAPIEE study. The risk was twice as high for persons with diabetes even after controlling for the sociodemographic factors and other lifestyle-related factors. This factor accounts for 23% of the population attributable risk of fatal cardiovascular event.

Results from the HAPIEE study further confirmed a protective effect of partnership on cardiovascular health. In the monitored age group (45–69 years), this can be explained mainly by ‘protective hypothesis’, which assumes that the presence of a partner provides certain psychological and social support, help during illness, help to cope better with stress situations, provides better and more stable economic situation, healthier and more responsible lifestyle and easier access to information about health and healthcare.^24^

The effect of obesity on CVD health was evaluated using the BMI, which still remains to be a significant predictor of both general and specific mortality.^25^ Our model monitored only the effect of obesity (ie, BMI ≥30 kg/m^2^ vs BMI <30 kg/m^2^) on CVD mortality. As an individually estimated factor, obesity increased the risk of fatal CVD by 62%. In the final model, the effect (HR) was 29%. In terms of PAF%, the obesity was only responsible for 11% fatal cardiovascular event.

The effect of total cholesterol level on the cardiovascular health was not found in our model. This could be caused by several facts. There was a high proportion of persons without a detected value of this marker among the cohort and especially among the deceased. Furthermore, precise results would be achieved, if the variable was included in the model as a continuous variable which was not possible due to data limitation (the high number of missing values treated as single category). Another fact that could have resulted in such result is the high prevalence of this factor in the population. Confirmation of the effect of a factor, when a large part of the population is affected, is usually very difficult.^26^ Nearly 76% of the HAPIEE cohort with analysed blood sample was detected with a higher cholesterol level (above 5 mmol/L). And finally, the total blood cholesterol level plays role in cardiovascular risk when cumulating in individual with other metabolically linked risk factors; the total blood cholesterol level is a more accompanying phenomenon than a single independent risk factor.

Another covariate without a confirmed effect on CVD health in our study was binge drinking. This variable is probably most influenced by subjective responses and consequently by the underestimation of consumption. On the other hand, similar results were presented by Malyutina *et al* 27 by data from the Multinational MONItoring of trends and determinants in CArdiovascular disease study in the Russian population. Risk of CVD death increased in persons falling into the category of really heavy alcohol consumers (160 g or more of pure alcohol), but occasional binge drinking did not increase cardiovascular risk. Malyutina *et al* 27 chose 160 g or more of pure alcohol to be the criteria for binge drinking. The analysis of the HAPIEE study used a more moderate criteria level of 100 g.

## Conclusion

This study based on HAPIEE cohort data suggests that estimated impact of main risk factors on cardiovascular health is largely consistent with the general finding about CVD risk factors; however, their manifestation (PAFs) is quite unique among the Czech population. Education had the largest impact on cardiovascular mortality among the Czech population. More than 50% of CVD death would be prevented if the whole population had the same risk values as the university educated population. Education is strongly linked not only to health but also to health determinants involving the health risky behaviour and preventative service use. Reducing disparities in health related to education should benefit from attention to cardiovascular health literacy.

**What is already known on this subject**

At the end of 1980s, rapid improvements in health condition were recorded and a late cardiovascular revolution took place in Czechia. This trend diverted Czechia from most of the Eastern European countries closer to Western European countries; however, the east–west mortality gap still persists in the case of cardiovascular diseases.

**What this study adds**

This study fills the gap in what is known about cardiovascular risk factors in Central Europe. Estimated impact of main risk factors on cardiovascular health is largely consistent with general finding about cardiovascular disease risk factors; however, their manifestation (PAFs) among the population is quite unique. Education had the largest impact on cardiovascular mortality among the Czech population. More than 50% of cardiovascular disease death would be prevented if the whole population had the same risk values as the highest educated population.
